# Progress in partially degradable titanium-magnesium composites used as biomedical implants

**DOI:** 10.3389/fbioe.2022.996195

**Published:** 2022-09-07

**Authors:** Jianping Wang, Zhifan Bao, Chenliang Wu, Song Zhang, Ningwei Wang, Qiang Wang, Zhe Yi

**Affiliations:** ^1^ School of Materials Science and Engineering, Shenyang University of Technology, Shenyang, China; ^2^ School and Hospital of Stomatology, China Medical University, Liaoning Provincial Key Laboratory of Oral Diseases, Shenyang, China; ^3^ School of Materials Science and Engineering, Northeastern-University, Shenyang, China

**Keywords:** titanium-magnesium composite, degradation, implant, mechanical property, galvanic corrosion

## Abstract

Titanium-magnesium composites have gained increasing attention as a partially degradable biomaterial recently. The titanium-magnesium composite combines the bioactivity of magnesium and the good mechanical properties of titanium. Here, we discuss the limitations of conventional mechanically alloyed titanium-magnesium alloys for bioimplants, in addition we summarize three suitable methods for the preparation of titanium-magnesium composites for bioimplants by melt: infiltration casting, powder metallurgy and hot rotary swaging, with a description of the advantages and disadvantages of all three methods. The titanium-magnesium composites were comprehensively evaluated in terms of mechanical properties and degradation behavior. The feasibility of titanium-magnesium composites as bio-implants was reviewed. In addition, the possible future development of titanium-magnesium composites was discussed. Thus, this review aims to build a conceptual and practical toolkit for the design of titanium-magnesium composites capable of local biodegradation.

## Introduction

Tissue injury is an unavoidable part of everyone’s existence. In some circumstances, tissue injury cannot heal itself, which means hard tissue repair components must be implanted to help the injured tissue heal (Zhang L. et al., 2019). Autologous bone grafts are without a doubt the greatest material for restoration (Rodriguez-Merchan, 2022), with no rejection and a high graft success rate. Nonetheless, due to the limited availability of materials and the difficulty of adjusting shapes and properties, it is difficult to apply them on a large scale. In order to overcome the limitations of self-bone grafting, research into synthetic bone repair materials is gradually gaining attention. And with the help of computer aided design and numerical simulations, synthetic bone repair materials have been vigorously developed (Zach et al., 2014). Metals, ceramics and polymers are commonly used as hard tissue replacement materials. Metallic materials play a role in most plastic surgery devices and dental implants, including temporary implants and permanent implants (Taddei et al., 2004; Chen and Liu, 2016). Nowadays many types of industrial metals are available, however, since bio-implants are implanted in the human body, only a few metals can meet the requirements for development as bio-implants. Conventional implants, which have been extensively used for orthopedic applications and are constructed of Ti-based alloys (Liu et al., 2019; Yan et al., 2019), stainless steel (Brooks et al., 2017; Teo et al., 2021), and cobalt-based alloys (Mas Ayu et al., 2019), will remain inside the body permanently after its implantation. Magnesium (Maier et al., 2020; Wu et al., 2021; Zeller-Plumhoff et al., 2021) are also classified as implants as biodegradable materials, in addition to the metals indicated above. In general, stainless steels and Co-based alloys suffer from a major biological drawback. For example, these two permanent alloys suffer from corrosion, which causes the liberation of allergenic/toxic Ni and Cr ions into bodily fluids. The release of these harmful ions will activate adverse inflammatory and immune responses *in vivo*, which becomes a primary shortcoming for long-term use in the biological surroundings (Wong and Man, 2018). Low density and high strength are advantages of titanium, and a dense oxide film will form on the surface of titanium and titanium alloys when titanium and titanium alloys come into contact with air or an oxygen-containing medium (Tang et al., 2018). Titanium alloys have been widely used in the field of biomedical applications due to their excellent biocompatibility and mechanical properties (Fan et al., 2021). Magnesium is a light metal with a density of 1.74 g/cm^3^, which is equal to the density of human bone (1.75 g/cm^3^) (Zhou H. et al., 2021). Due to their superior mechanical compatibility, biocompatibility, and degradability, magnesium and magnesium alloys have a wide range of biomedical uses. Magnesium is one of the body’s macronutrients and is involved in a number of metabolic activities. The most important of these is that it promotes calcium deposition, which is good for bone formation (Makkar et al., 2018).

The respective shortcomings of titanium and magnesium materials limit their widespread use. The disadvantage of titanium is that its Young’s modulus (E), although lower than that of other metals, is still somewhat different from that of human bone, which inevitably leads to “stress shielding” (Chen and Thouas, 2015). As a result, past studies have focused on reducing the E of biomedical titanium materials. β-phase titanium has been found to be a phase with a low E (Zhou L. et al., 2021), so increasing the content of β-phase in titanium alloys has become a significant focus of research (Koizumi et al., 2018; Aguilar et al., 2019; Pellizzari et al., 2020). Titanium alloys with E as low as 33 GPa have been developed (Hao et al., 2007). In addition to increasing the content of the β-phase to reduce the E, the introduction of a porous structure is also considered as an excellent way to reduce the E (Khodaei et al., 2018). Due to its unique pore design, porous titanium deforms more than bulk materials when subjected to the same force. As β-phase titanium alloys require the addition of a number of β-phase stabilizing elements (Qazi et al., 2005), the addition of these heavy elements increases the risk of cytotoxicity caused by release of metal ions (Yu et al., 2015). Furthermore, titanium alloys enhance the likelihood of a patient’s allergy after implantation (Fage et al., 2016). Weighing the pros and cons, porous titanium is considered to be an effective way to avoid the “stress shielding” phenomenon. By altering the porosity of porous titanium, the modulus of elasticity may be accurately regulated, allowing it to conform exactly to that of human bone tissue. The presence of a porous structure provides not only a channel for the movement of nutrients, but also space for tissue cell proliferation (Meenashisundaram et al., 2020). However, as titanium is a biologically inert material, certain surface modifications to the surface of the metal titanium are required to enable it to induce the formation of human bone tissue (Su et al., 2020). The surface modification treatment would make the preparation process more complicated and increase the production cost, which limits the wide application of porous titanium. The main problem faced by magnesium and magnesium alloys when implanted into the human body is their rapid degradation rate (Bütev Öcal et al., 2020), which can lead to an increase in pH in local areas of the tissue and a series of problems caused by rapid hydrogen precipitation. Rapid corrosion rates will eventually lead to premature loss of mechanical properties of magnesium and magnesium alloys. In this regard, reducing the degradation rate *in vivo* is a major problem for Mg based materials. High purity Mg alloy has a lower content of impurity elements, and although the degradation rate is lower than that of commercially pure Mg, the mechanical strength of high-purity magnesium is so low that it is not suitable for biological implants. For magnesium, alloying is an important step to improve its corrosion resistance. Magnesium alloys such as AZ91D, WE43 and ZK60 are considered to last longer than commercially pure magnesium in the human body (Witte et al., 2005; Asri et al., 2017).

In view of the inherent drawbacks of the Ti-based and Mg-based materials, titanium-magnesium composites for partially biodegradable implants have drawn the attention of researchers in recent years, which might circumvent the disadvantages of the permanent and non-permanent implants currently in use. Initially, scholars began to research the feasibility of titanium-magnesium composites as orthopedic load-bearing implants (Li et al., 2015). Subsequently, the mechanical and fatigue properties of titanium-magnesium composites have been shown to be comparable to Grade 4 Ti (Balog et al., 2017). Therefore, titanium-magnesium composites are also a viable option for dental implants. In addition, titanium-magnesium composites have also been developed for use in bioinspired fish scales (Liu X. et al., 2021). In summary, titanium-magnesium composites have a wide range of application prospects.

This paper presents an overview of recent research and developments of titanium-magnesium composites used as biomedical materials. It can be divided into three main sections, beginning with preparation of titanium-magnesium composites, outlining three suitable methods for the preparation of titanium-magnesium composites. This section is followed by summarizing mechanical properties of various titanium-magnesium composites and discussing the effects of all phases and alloying elements in titanium-magnesium composites on mechanical properties. The third section considers effect of material ingredient, volume ratio, and other factors on corrosive properties, describing the status of current titanium-magnesium composites used as biomaterials and their limitations. Overall, efforts have been made to reveal the latest scenario of biomedical titanium-magnesium composites. The goal of this review is to create a conceptual and practical toolset for the design of titanium-magnesium composites that can degrade locally.

## Processing methods

**FIGURE 1:**
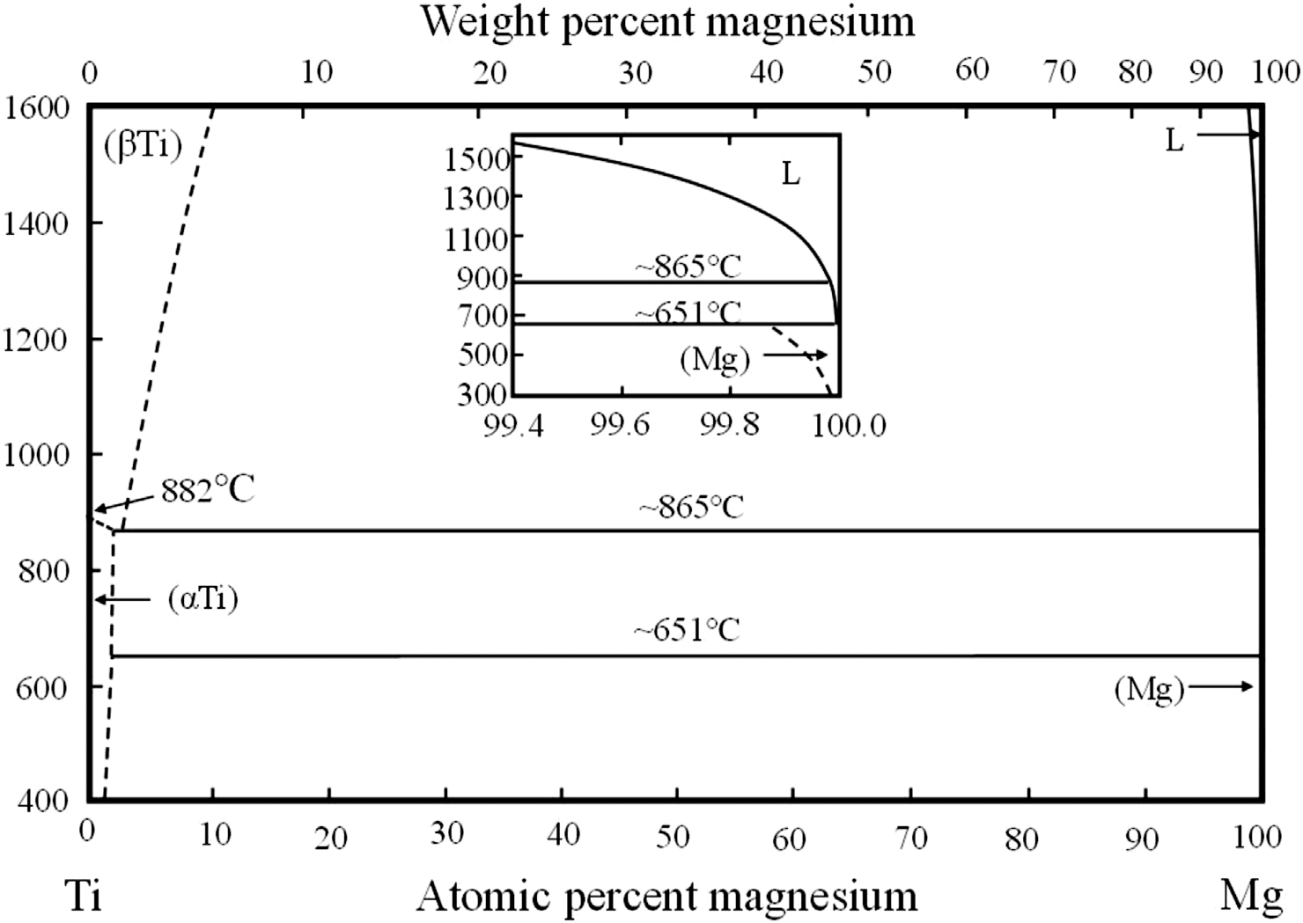
Titanium-magnesium binary phase diagram.

Alloying Ti with Mg is a common strategy for preparing titanium-magnesium alloy. There are two inherent obstacles in the traditional method of preparing titanium-magnesium alloys. On the one hand, titanium is a high temperature resistant metal, with a melting point of 1668°C, which is much higher than the boiling point of magnesium (1070°C). On the other hand, magnesium only acts as solute atoms in the titanium matrix. It was reported that the solid solubility of magnesium in titanium at room temperature is only 0.9 at. % and that of titanium in magnesium is 0.02 at. % (Yao et al., 2022). Figure 1 shows the phase diagram of titanium-magnesium binary alloy. As can be seen obviously, titanium and magnesium cannot be mutually soluble in the full composition range, and there are no eutectic phases or stable intermetallic compounds in the temperature of 600–1000°C. Owing to the significant difficulty of alloying between titanium and magnesium, producing titanium-magnesium alloys by traditional melting processes is nearly impossible. However mechanical alloying can extend the solid solubility of solid atoms and is considered as an effective method to increase the solid solubility of magnesium in titanium (Liang and Schulz, 2003). Cai et al. (2018) carried out ball-milling tests through titanium powder and magnesium powder for 50 h, and then compacted in a cubic-anvil press under a pressure of 4 GPa at RT. After annealed for 1 h, they prepared the Mg-1.5 at.% Ti alloy. In addition, Liang et al. (2021) have also prepared titanium-magnesium alloy with Mg content ranging from 0.312 wt.% to 2.5 wt.% by mechanical alloying method. According to their study, when the Mg content is low, Mg could improve the mechanical strength of Ti due to the co-strengthening effects of precipitation and solid solution. When the Mg content reaches 1.25 wt.%, it will be detrimental to the mechanical properties of the material. As a consequence, mechanical alloying can effectively enhance the solid solubility of magnesium in titanium. Nevertheless, the magnesium content in titanium is still too low to exert obvious influence on the titanium-magnesium alloys. Due to the low solid solution of magnesium in titanium, titanium-magnesium composites prepared by infiltration casting, powder metallurgy and hot press forging have drawn scholars’ attention.

### Infiltration casting

**FIGURE 2:**
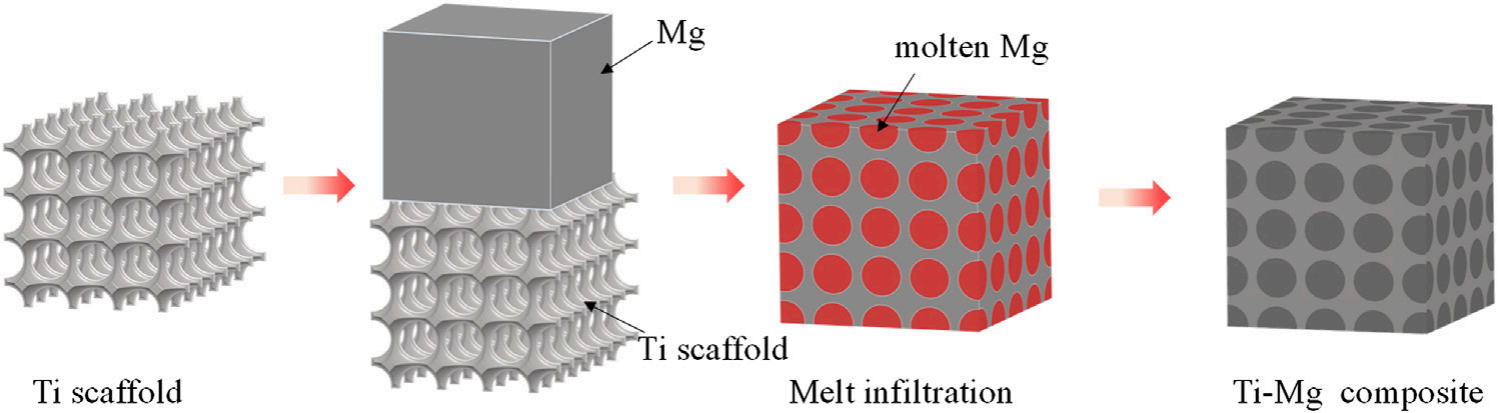
The schematic diagram of preparing titanium-magnesium composites by infiltration casting.

Infiltration is a liquid-state fabrication method, in which a porous preform is impregnated in a molten matrix metal to fill the pores. Mechanical winding, powder metallurgy, 3D printing and other techniques were used to prepare porous titanium, and then molten magnesium was used to fill the pores of the porous titanium, resulting in a titanium-magnesium composite (Li et al., 2015; Esen et al., 2016; Meenashisundaram et al., 2020). The schematic diagram of preparing titanium-magnesium composites by infiltration casting is shown in Figure 2. Li et al. (2015) twisted titanium wire into a spiral form, which was then stretched and woven into a 2D mesh. The mesh was formed into a 3D material and pressed in a mold to make the cylindrical porous titanium (p-Ti) preform. Subsequently, molten Mg was infused into the porous titanium, and the titanium-magnesium composite material was formed after cooling to room temperature. According to their research, the stiffness of p-Ti/Mg composites is greatly improved compared to entangled titanium, but only comparable to that of pure magnesium. The strength of p-Ti/Mg needs to be further improved as a biomedical implant. However, its residual strength after degradation is favorable for biomedical applications. Esen et al. (2016) obtained porous structures of Ti and Ti-6Al-4V skeletons by loose powder sintering and obtained titanium-magnesium composites with better mechanical properties by capillary penetration of molten magnesium. Meenashisundaram et al. (2020) 3D inkjet printing technology to mix titanium powder and polyvinyl alcohol to prepare porous titanium parts, followed by pressureless infiltration of molten magnesium to obtain titanium-magnesium composites. 3D printing enables faster and more cost-effective manufacture of net shapes for biomedical implants that meet patient needs and improves precision, fit and load distribution, and is also considered an excellent technology for machining and preparing titanium alloys (Yi et al., 2021). In summary, the advantages of titanium-magnesium composites prepared by infiltration casting method are simple operation, low manufacturing cost, and large-scale industrial production. The disadvantage is that the bonding strength of the titanium-magnesium interface is low, and the wettability and fluidity of the metal solution need to be further improved.

### Powder metallurgy

**FIGURE 3:**
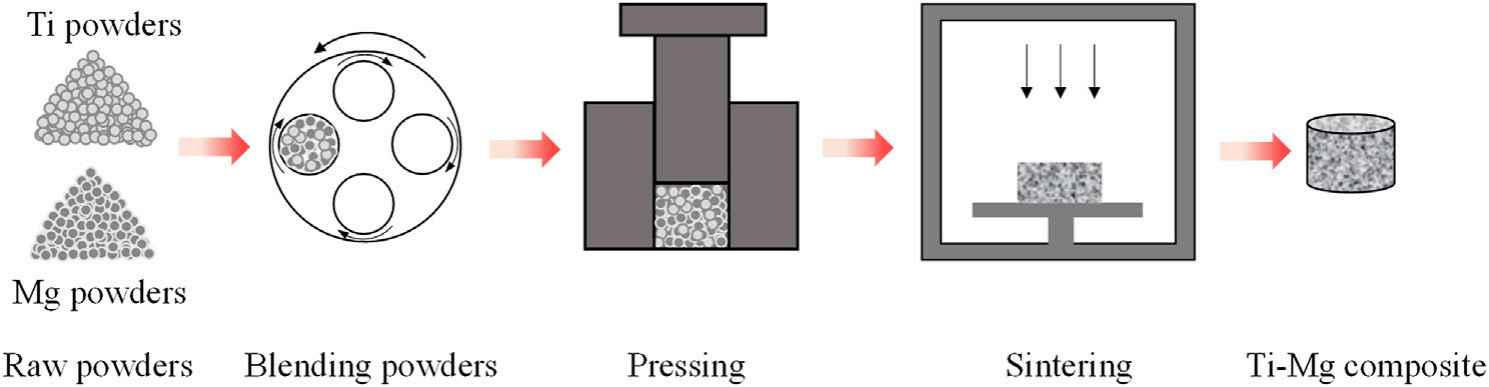
The schematic diagram of preparing titanium-magnesium composites by powder metallurgy.

Powder metallurgy preparation of titanium-magnesium composite materials is a technology that uses titanium powder and magnesium powder as raw materials, which are mixed, pressed and sintered by ball milling. The schematic diagram of preparing titanium-magnesium composites by powder metallurgy is shown in Figure 3. With relatively low powder metallurgical sintering temperature, titanium-magnesium composites are easy to operate, with low energy consumption and high precision. Sintering is the most critical part of powder metallurgy and has a direct impact on the mechanical properties of the material. According to the different sintering processes, powder metallurgy can be divided into spark plasma sintering (SPS), microwave sintering, atmosphere sintering, etc. SPS is a fast, low-temperature, energy-saving and environmentally friendly new material preparation technology (Hu et al., 2020). Ouyang et al. (2020) prepared titanium-magnesium composites by SPS technique through irregularly shaped commercial titanium powders and x vol% (x = 10, 20 and 30) Mg-3Zn powders as raw materials, and the relative density values of titanium-magnesium composites are all higher than 98%. However, the content of Mg is low in the titanium-magnesium composites prepared by SPS. Therefore, the distribution of Ti phase in the prepared composites was continuous, while the distribution of Mg-3Zn phase was uniform rather than discontinuous, and these Mg-3Zn did not form continuous channels but independent pores after degradation. The MgO was also found according to XRD analysis, while the effect of MgO on the properties of the titanium-magnesium composites has not been investigated in depth. In order to avoid generation of oxides during the preparation process, Ibrahim et al. (2020) successfully prepared Ti-xMg (x = 0, 12, 17 and 24 vol.%) at a temperature below 450°C, and did not find the aggregation of oxygen elements at the titanium-magnesium interface. The powder metallurgy method can prepare titanium-magnesium composites quickly and efficiently. However, powder metallurgy also has many serious problems. For example, the oxidation of raw materials during the sintering process and the lack of metal compound phase formation at the interface between titanium and magnesium phases lead to poor interfacial bonding strength.

### Hot rotary swaging

**FIGURE 4:**
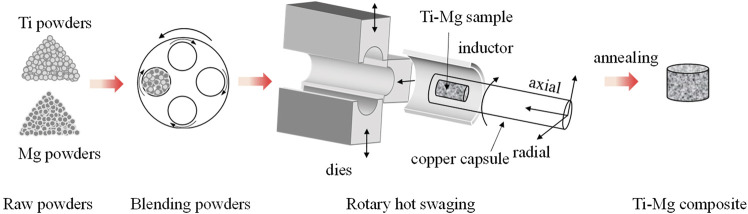
The schematic diagram of preparing titanium-magnesium composites by hot rotary swaging.

**FIGURE 5:**
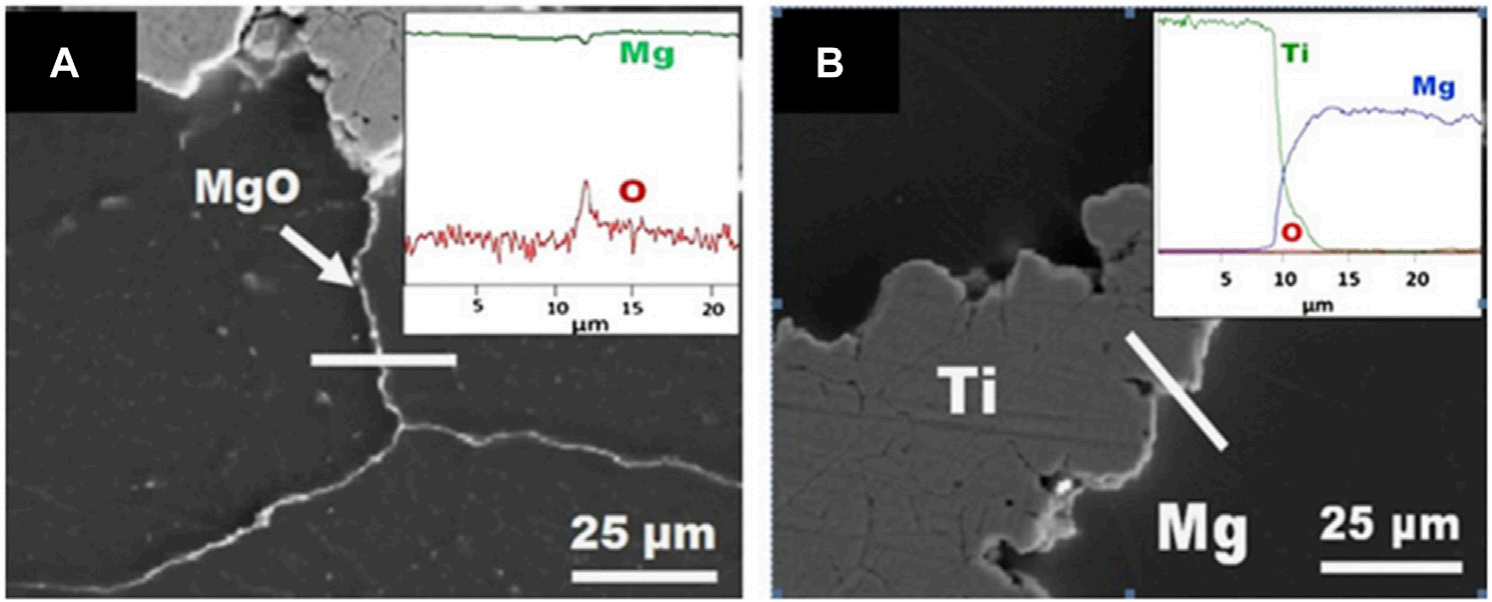
SEM micrograph of titanium-magnesium composites containing 80 vol% Mg **(A)** MgO layers and EDS line scan taken along white line and **(B)** titanium-magnesium interface and EDS line scan across titanium and magnesium (Esen et al., 2013).

Hot rotary swaging is an incremental shaping process, which is commonly used to modify the cross-sections of rotationally symmetric metal objects such as rods, tubes, and wires, as well as to link various materials or components. The hot rotary swaging procedure reduces grain-size in metallic materials, and effectively improves the mechanical properties of the material, and hot rotary swaging has been used for various demanding materials and alloys (Chi et al., 2019; Kunčická et al., 2020; Strunz et al., 2020). The schematic diagram of preparing titanium-magnesium composites by hot rotary swaging is shown in Figure 4. Esen et al. (2013) used titanium and magnesium powder as raw materials, the titanium-magnesium composites with Mg volume fractions of 50, 60, 70 and 80% were prepared by hot rotary forging technology, followed by a 1-h annealing treatment at 600°C aiming at obtaining homogeneous microstructure of the material. The SEM micrographs of titanium-magnesium composites containing 80 vol% Mg are shown in Figure 5. Figure 5A illustrates the presence of oxygen enrichment between Mg-Mg, which indicated the presence of a very thin oxide layer between Mg-Mg. As shown in Figure 5B, the elemental oxygen content between titanium-magnesium was only slightly increased because MgO was present only on the Mg powder of the original material. The titanium-magnesium composite prepared by hot press forging method has high density and good plasticity, but it should pay attention to the various reactions of magnesium powder with oxygen during the processing.

As a consequence, it is challenging to produce titanium-magnesium composite materials, and it is even harder to create them using conventional techniques. The three processes listed above had successfully prepared titanium-magnesium composites and each method has its own advantages. The titanium-magnesium composites prepared by powder metallurgy method will inevitably encounter magnesium volatilization during the preparation process, so the manufactured titanium-magnesium composites may differ from the initial design of the titanium-magnesium content. The titanium-magnesium composites prepared by powder metallurgy method often have low magnesium content, and the volatilization of magnesium will result in the presence of holes in the prepared composites. Hot rotary swaging technique can produce titanium-magnesium composites with a high magnesium content. However, its preparation process and subsequent heat treatment process inevitably react with oxygen to generate oxides such as MgO, which may adversely affect the properties of the composites. Therefore, it is not the best way to produce titanium-magnesium composites. The infiltration casting method meets the requirements of mass production, and can also control the total porosity, pore size and pore distribution of porous titanium scaffolds through 3D printing and other technologies to produce titanium-magnesium composites with specific structures to replace bone in different parts. In addition to the preparation techniques mentioned above, various cutting-edge technologies have been applied to the production of alloys with low miscibility. For instance, due to the extremely quick cooling rate, laser rapid solidification is also known as a potential method for creating metastable structures and alloys with low miscibility.

## Mechanical properties

Ti and Ti-6Al-4V are commonly used as support structures for the preparation of titanium-magnesium composites (Wang et al., 2011; Esen et al., 2020). Commercially-pure Ti (CP-Ti) as an α titanium alloy, only the CP-Ti with grade 4 is used for dental applications or forfabrication of porous coatings rather than joint implants due to its low mechanical strength at room temperature (Chen and Thouas, 2015). Ti-6Al-4V is the most widely used bio-applicable Ti-based α+β alloy. The properties of Ti-6Al-4V can be optimized by adjusting the volume fraction of the α and β phases through different heat treatments due to the presence of both, an α stabilizer (Al) and a β stabilizer (V) (Vrancken et al., 2012). Compared with CP-Ti, Ti-6Al-4V exhibits better mechanical properties. However, the V and Al elements added in Ti-6Al-4V are considered to be cytotoxic, especially that Al ions may depress bone growth and even exert a potential danger of Alzheimer’s disease (Tamilselvi et al., 2006; Li et al., 2014). Currently, research and development in the field of Ti-based alloys is focused on two main objectives. The initial goal is to change the chemical compositions of the alloys in order to replace problematic components. The second goal is to create alloys with characteristics that are as near to bone as possible (Kunčická et al., 2017). The β-type titanium alloys containing more β-phase stabilizers (Mo, Zr and Ta) possess lower modulus of elasticity and higher toughness than Ti-6Al-4V bulk (Zhang Y. S. et al., 2019). The corrosion resistance of β-type titanium alloys in the human body is also higher than that of (α + β) titanium alloys such as Ti-6Al-4V (Carman et al., 2011). The incorporation of these rare metal elements makes the preparation of β-titanium alloys complex and increases the cost of raw materials (Narita et al., 2012). The design of low-cost β-titanium alloys has been in progress and will be used on a large scale for biomedical applications in the foreseeable future (Gepreel and Niinomi, 2013). Most titanium-magnesium composites currently use pure titanium as the raw material due to safety and cost-effectiveness considerations.

**FIGURE 6:**
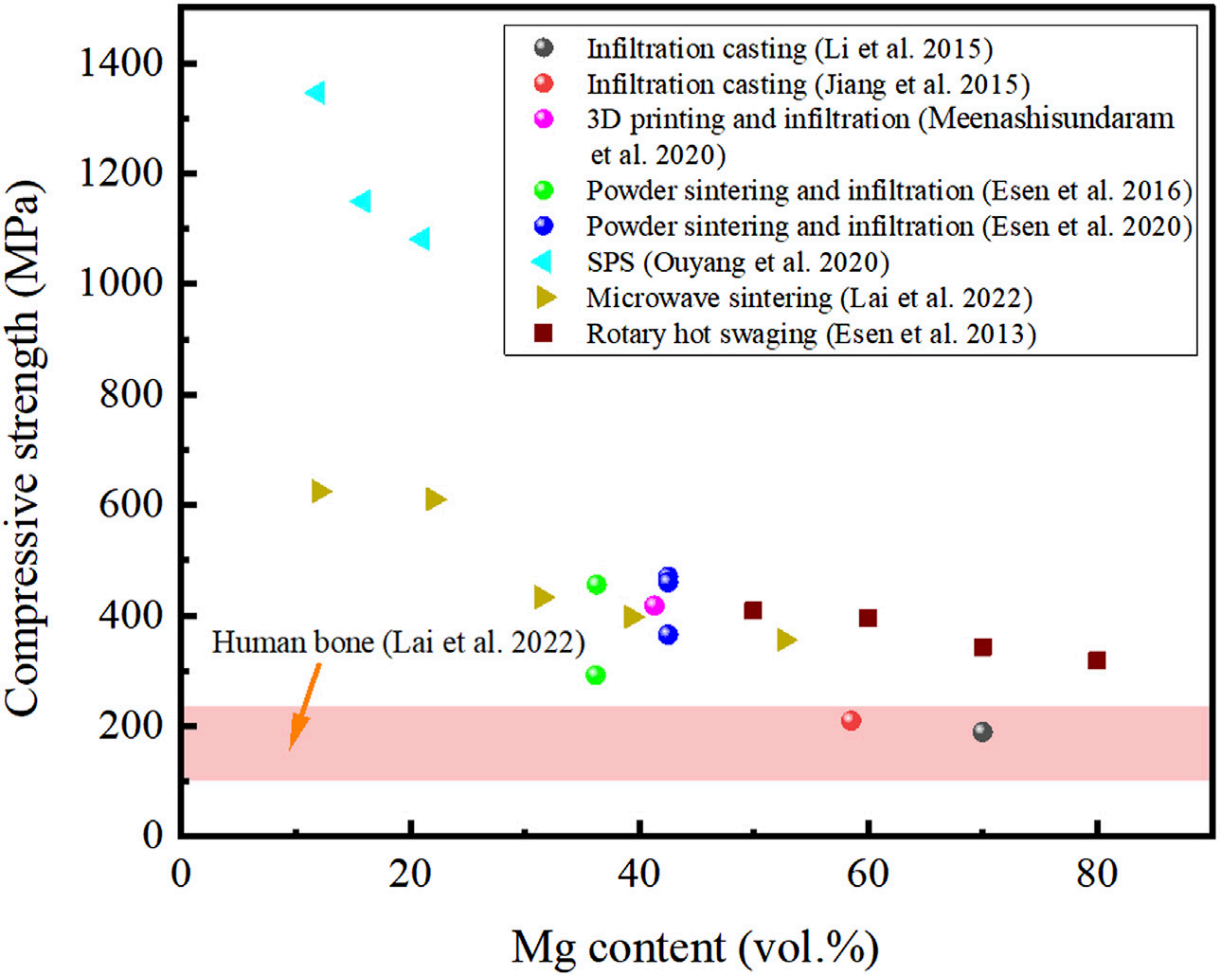
The UCS of human bone and titanium-magnesium composites as a function of Mg content prepared by different preparation processes.

The mechanical properties of titanium and titanium alloys with porous structures are greatly reduced compared to the bulk titanium and titanium alloys. However, the mechanical properties of the composite can be correspondingly improved with magnesium completely filling the internal pores, while it is still far less than that of bulk titanium and titanium alloys. According to recent study, titanium-magnesium composites with specific spatially aligned structures can promote effective stress transfer, delocalize damage and arrest cracking, thereby bestowing improved strength and ductility (Zhang et al., 2022). In addition, extensive studies revealed that the mechanical properties of the titanium-magnesium composite are sufficient to meet the performance requirements of human implants. The human skeleton mainly plays a load-bearing role in the human body, so the study of ultimate compressive strength (UCS) is more relevant compared to the ultimate tensile strength. Figure 6 shows the UCS of human bone and titanium-magnesium composites as a function of Mg content prepared by different preparation processes. It is noticed that the UCS for all titanium-magnesium composites in Figure 6 is higher than that of human bone. Since the mechanical properties of titanium are higher than those of magnesium and the interface between titanium and magnesium cannot be alloyed, the UCS of titanium-magnesium composites prepared by the same preparation process decreases with increasing Mg content. As the E of titanium is higher than that of magnesium, Ti will be subjected to a higher force when the titanium-magnesium composites withstand external forces. Therefore, cracks preferentially appear in the Ti phase or at the titanium-magnesium interface where the bond strength is not high. It can also be found that Ti tends to exhibit a ductile fracture mode, while magnesium exhibits a typical brittle fracture (Ouyang et al., 2020). Ibrahim et al. (2020) compared the mechanical properties of two composites prepared from the same magnesium powder and different titanium powders, and found that the titanium-magnesium composites prepared from hydrogenated-dehydrogenated titanium powder showed an improvement in yield strength and ultimate tensile strength compared to that prepared from plasma atomized titanium powder. This may be because the hydrogenated-dehydrogenated titanium powder surface has a larger concentration of TiO_2_, and the Ti-O solid solution may act as a strengthening agent, resulting in stronger mechanical characteristics. However, the load transfer from Ti to Mg during straining, may be hampered by the presence of TiO_2_ dispersoids at the contact. Esen et al. (2016) prepared titanium-magnesium composites using Ti and Ti-6Al-4V powders with Mg, and the mechanical properties of the obtained Ti-6Al-4V/Mg were superior to those of Ti/Mg composites. In addition, according to the research of Jiang et al. (2018), the mechanical properties of titanium-magnesium composites prepared with an average diameter of 100 μmTi powders are better than those prepared by 230 μm Ti powders. This is due to stress concentration in the sintered neck region of Ti particles, leading to pre-fracture. And smaller Ti particles means more sintering neck in the same volume. Although the contribution of magnesium to the composites is not very obvious, the mechanical properties of titanium-magnesium composites prepared with AZ91 and WE43 are higher than those prepared with pure Mg (Esen et al., 2020). In addition to the use of different raw materials to optimize the mechanical properties of the titanium-magnesium composites, a unique preparation process can also improve the mechanical properties of the composites. Lai et al. (2022) obtained titanium-magnesium composites by microwave sintering and found that the UCS of the titanium-magnesium composites were greatly improved compared with other preparation processes under the same Mg content. Ouyang et al. prepared titanium-magnesium composites by SPS, aiming at improving the mechanical properties. Due to the influence of the nanograin size microstructure produced by the SPS process, the UCS of the titanium-magnesium composites was as high as 1346.3 MPa (Ouyang et al., 2020).

**FIGURE 7:**
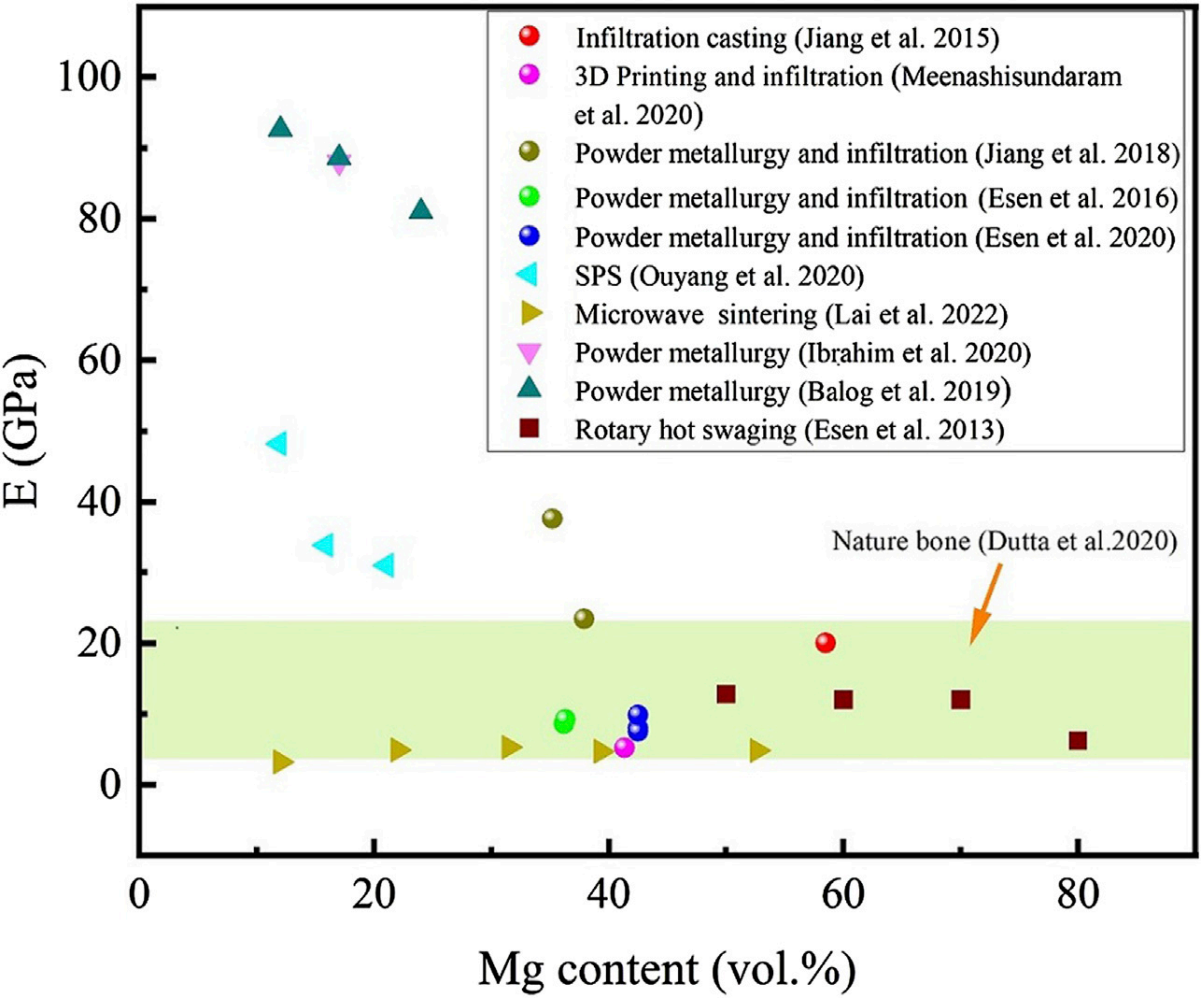
The E of nature bone and titanium-magnesium composites as a function of Mg content prepared by different preparation processes.

Figure 7 shows the E of nature bone and titanium-magnesium composites as a function of Mg content prepared by different preparation processes. Overall, the E of the titanium-magnesium composites showed a decrease tendency with the increase of the volume fraction of Mg. According to the study reported by Balog et al. (2019), if the Mg component (E = 45 GPa) is expected to contribute to elastic load transmission following a simple mixing rule, the E of the Titanium-magnesium composite should drop at a rate of 0.55 GPa per one vol% Mg. Another boundary condition is that if Mg does not contribution to elastic load transfer, the E of the titanium-magnesium composite should theoretically drop at a rate of 0.99 GPa per one vol% Mg. According to the results of their study show that the volume share of Mg share decreases by 0.78 GPa for every one vol% increase in the volume of Mg. This indicates that the interface between titanium and magnesium is often not an ideal metallurgical bonding and has a limited contribution to the load transfer in the elastic region. Theoretically, the E of titanium-magnesium composites should decrease with the increase of Mg content between the E of pure titanium and pure magnesium, but not lower than that of pure magnesium. The volume percentage of Mg in the microwave sintered titanium-magnesium composites is not very high, but its E is as low as below 10 GPa (Lai et al., 2022). This phenomenon can be attributed to the fact that the material prepared by microwave sintering will volatilize part of the Mg during processing because of the faster heating temperature, which leads to more pores in the composite. According to research of Rivera-Salinas et al. (2020), the holes in the composite materials significantly reduce the E of the overall material. The E of titanium-magnesium composites should theoretically be between Ti (E = 110 GPa) and Mg (E = 45 GPa), and decrease correspondingly with the increase of Mg content. If the dense density of the composite is 100%, the E of titanium-magnesium composites will not be lower than 45 GPa. However, the E of many titanium-magnesium composites were found to be lower than 45 GPa. These composites with low E may be due to the imperfect preparation process, which leads to the existence of pores in the composites and makes the material bonding not tight enough, thus the E of titanium-magnesium composites may be lower than that of pure magnesium. The composition of titanium-magnesium composites can be varied, and the strength and E can be adjusted in a wide range by selecting the appropriate titanium-magnesium composition ratio according to different parts of the skeleton.

## Degradation performance

### Degradation behavior

**FIGURE 8:**
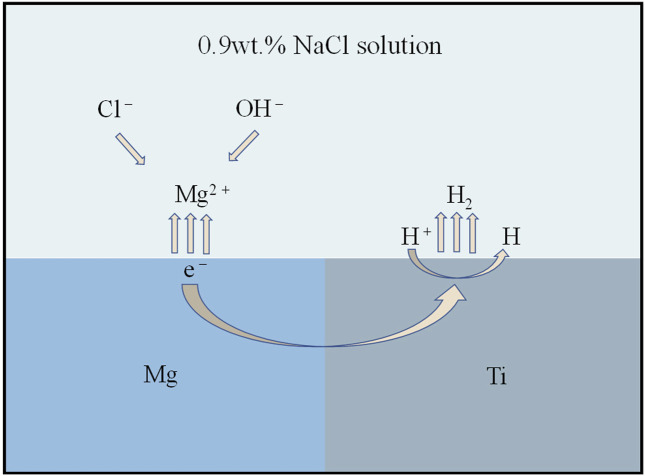
The schematic diagram of galvanic corrosion of titanium-magnesium composites.

Since a dense oxide film forms on the surface of titanium and titanium alloys, it effectively prevents further corrosion of the material by the solution (Zhou X. et al., 2021). Mg is more active and will degrade in human body. Therefore, the corrosion behavior of titanium and magnesium composites in these solutions, such as Hanks’ solution, Ringer’s solution and SBF solution, is mainly dominated by the degradation of Mg. The pH, electrolytes, amino acids and proteins of these solutions are as close as possible to the actual human environment (Bahraminasab et al., 2019; Heywood et al., 2019; Tiyyagura et al., 2020). Ti plays an important role in the degradation process of Mg. The electrochemical potential of the standard hydrogen electrode of Mg is −2.37 V, and that of the standard hydrogen electrode of Ti is −1.63 V. Because of the potential difference between Ti and Mg, galvanic corrosion occurs when Ti and Mg are in direct contact. Galvanic corrosion is an electrochemical process in which the reduction potential of an ion usually determines its ability to gain electrons and form a solid metal. The “active” metal with the lower reduction potential of the two metals will be corroded (anode). In comparison, the “noble” metal with the higher reduction potential will be deposited (cathode) (Koh et al., 2015). Thus, when metals with different reduction potentials pair, micro-movement and pitting will occur. The schematic diagram of the galvanic corrosion of the titanium-magnesium composite is shown in Figure 8. The galvanic corrosion reaction of titanium-magnesium composites is as follows (Wu et al., 2013).

$$\text{Anodic reaction: Mg}\to {\text{Mg}}^{2+}+2e^{-}$$ (1)

$${\text{Cathodic reaction: }2\text{H}}_{2}\text{O}+2e^{-}\to {\text{H}}_{2}↑+{2\text{OH}}^{-}$$ (2)

$$\text{Overall reaction: Mg}+{2\text{H}}_{2}\text{O}\to \text{Mg}{\left(\text{OH}\right)}_{2}↓+{\text{H}}_{2}↑$$ (3)

**FIGURE 9:**
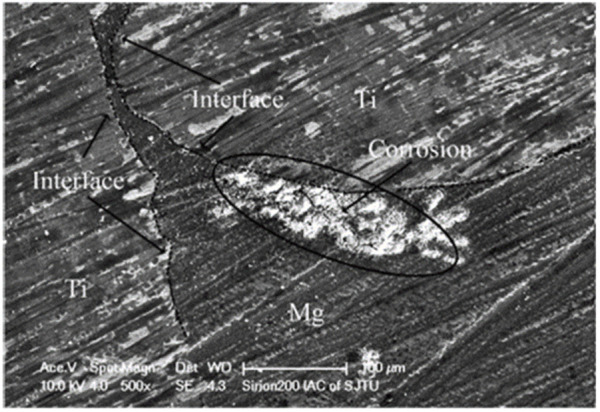
Surface morphology of the p-Ti/Mg composite after immersion in Hanks’ solution for 10 min (Li et al., 2015).

Li et al. (2015) compared the corrosion rates of p-Ti/Mg and pure Mg in Hanks’ solution through hydrogen precipitation experiments. It was found that the hydrogen evolution rates of p-Ti/Mg with a duplex microstructure were much higher than that of pure Mg. A maximum value of 1.49 ml/cm^2^ h was reached at 52 h. Figure 9 shows the traces of galvanic corrosion observed for titanium-magnesium in Hanks’ solution. It can be seen from Figure 9 that the Mg near the Ti phase is preferentially corroded, while the Mg farther away from the Ti corrodes to a lesser extent. Although the electrochemical differences between titanium and magnesium were minimal, the less electronegative magnesium was sacrificed as the anode, thus accelerating the corrosion of magnesium. The magnesium around the titanium wire was rapidly corroded away, causing the magnesium substrate further away from the wire to an “island structure”, thus increasing the contact area between the magnesium and the solution and further accelerating the corrosion of magnesium. The electrocoupling corrosion will make the corrosion rate greatly accelerated, so how to reduce or avoid the electrocoupling corrosion in titanium-magnesium composites has become the focus of the research scholars. According to the study of Esen et al. (2013), it was found that the titanium-magnesium area ratio on the surface of titanium-magnesium composites will affect the degree of galvanic corrosion occurrence. When the surface area of Mg phase in the composite is smaller than that of Ti, a large cathode and small anode corrosion type will occur, and this corrosion mode will lead to the rapid transfer of electrons generated by the reaction of Mg with the solution through the Ti phase and generate hydrogen gas with the hydrogen ions in the reducing solution. When the area of Mg phase is much larger than that of Ti phase, a large anode and small cathode corrosion type will occur. And this corrosion mode is thought to slow down the rate of Mg corrosion rate, so the relatively small cathode area will lead to saturation of electron transfer, electrons cannot pass through the limited Ti timely reduction of hydrogen ions, so that the anode magnesium corrosion is mitigated. In addition, Esen et al. (2016) also found that reducing the potential difference between the two metals by alloying can also significantly attenuate the galvanic corrosion in titanium-magnesium composites. They found that the corrosion rate of Ti-6Al-4V/Mg composites was lower than that of Ti/Mg composites because Ti, by alloying with Al and V, effectively changed the electrochemical potential of Ti, thus alleviating the galvanic corrosion between the two metals in the composites. Unlike Ti, the alloying of Mg should be kept cautiously, because the electrochemical potential between different phases and impurities of Mg alloy also produces potential difference, and unreasonable alloying may lead to the cathodic center of Mg alloy itself, which in turn will accelerate the corrosion rate of Mg phase (Song and Atrens, 2003). Truncating the electron transfer between metals is considered to be the most effective method to inhabit electrocoupling corrosion. During the preparation of titanium-magnesium composites, a layer of MgO film will be produced at the interface of titanium and magnesium, this may adversely affect the bond strength of the material, but the continuous MgO layer will make an insulating layer between Ti and Mg, thus blocking the electron transfer channel, avoiding the occurrence of galvanic coupling corrosion and changing the corrosion response of the material, which can effectively reduce the corrosion rate of composite materials (Esen et al., 2013; Ouyang et al., 2020).

Galvanic corrosion is the main form of corrosion in the first stage of titanium-magnesium composites, with the degradation of Mg, a layer of Mg (OH)_2_ will be formed on the surface of the composite. Theoretically, this layer of Mg (OH)_2_ can hinder the further reaction between the internal Mg and the solution (Lai et al., 2022), but it is found that Mg (OH)_2_ only has a protective effect when the pH is higher than 11.5 (Tsakiris et al., 2021). And as a human implant, it tends to induce inflammation in the tissues around the implant at the early stage of implantation, thus creating an acidic environment (Liu Y. et al., 2021), which is unfavorable to magnesium hydroxide. In addition, it is found that when the chloride ion in the solution exceeds 30 mmol/L, magnesium hydroxide will be gradually eroded by chloride ion and further transformed into the more soluble MgCl_2_ (Zhao et al., 2016), and there is still abundant chloride ion in human body fluid. It will gradually erode this protective film of magnesium hydroxide, so that it can expose the Mg inside the matrix and promote the further dissolution of Mg. The specific reaction is as follows.

$$\text{Mg}{\left(\text{OH}\right)}_{2}+{2\text{Cl}}^{-}\to {\text{MgCl}}_{2}+{2\text{OH}}^{-}$$ (4)

$$\text{Mg}+{2\text{Cl}}^{-}\to {\text{MgCl}}_{2}+{2\text{e}}^{-}$$ (5)

Soluble MgCl_2_ is converted to magnesium phosphate by a series of reactions in a human environment rich in various ions or in a solution that mimics the human environment. Magnesium phosphate is then further reacted to produce hydroxyapatite. These insoluble apatite components are predicted to act as nucleation sites for the inward growth of new bone after the implantation process, thereby improving bio-compatibility and osseointegration standards (Heywood et al., 2019; Zhou H. et al., 2021). The reactions are follows.

$${\text{HPO}}_{4}^{2-}+{\text{OH}}^{-}\to {\text{PO}}_{4}^{3-}+{\text{H}}_{2}\text{O}$$ (6)

$$\begin{array}{l} 3{\text{Mg}}^{2+}+{2\text{PO}}_{4}^{3-}\to {\text{Mg}}_{3}{\left({\text{PO}}_{4}\right)}_{2}↓ \end{array}$$ (7)

$${10\text{Ca}}^{2+}+{6\text{PO}}_{4}^{3-}+{2\text{OH}}^{-}\to {\text{Ca}}_{10}{\left({\text{PO}}_{4}\right)}_{6}{\left(\text{OH}\right)}_{2}↓$$ (8)

**FIGURE 10:**
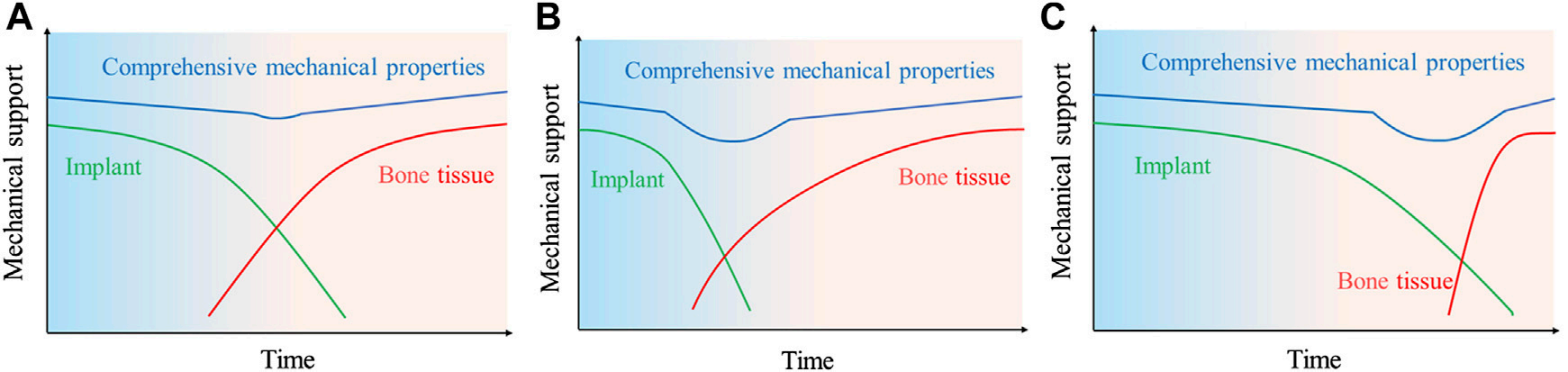
Schematic diagram of the mechanical support match between bone repair scaffold and tissue; green: red curves of scaffold implantation *in vivo* degradation, tissue repair at bone defect site; **(A)** ideal condition; **(B)** too rapid scaffold degradation; **(C)** too slow scaffold degradation.

Dynamic potential polarization testing is considered to be the most direct electrochemical method to evaluate *in vitro* corrosion processes. The corrosion potential and corrosion current of the material can be obtained by Tafer extrapolation. In Table 1, the corrosion potentials and corrosion currents of titanium-magnesium composites with different compositions are given. It can be seen that the corrosion potential of titanium-magnesium composites decreases with increasing Mg content, and the corrosion current increases rapidly with increasing Mg content. However, according to the research of Ouyang et al. (2020), the corrosion rate of Ti-30 Mg in SBF solution is higher than that of Ti-20 Mg. Since Mg agglomerates with increasing content during SPS preparation, the aggregation of Mg reduces the contact area between Mg and Ti phases, thus weakening the galvanic corrosion between Ti and Mg. Mg^2+^ concentrations of Ti-20 Mg grow quicker than those of Ti-10 Mg and Ti-30 Mg in the first 3 days, according to a follow-up study by Ouyang et al. (2020), Mg^2+^ concentrations are high in the cathode area, generating an ion cloud that obstructs the electrode process. Traditionally, the corrosion resistance of the materials is evaluated according to the grades in Table 2. According to the classification in Table 2, the corrosion resistance of titanium-magnesium composites is within the class 0–3. For magnesium and magnesium alloys, the poor corrosion resistance means that the rate of degradation is too fast and may face rapid failure of the implant, but for titanium-magnesium composites, due to the stable presence of titanium, even if magnesium degrades rapidly after implantation, it will not cause the titanium-magnesium composite to lose its proper mechanical support. Therefore, it is not comprehensive to evaluate whether titanium-magnesium composites have the possibility of implantation into human body only by the rate of degradation. The bone repair process in human implants consists of three phases: the inflammatory phase, the repair phase and the remodeling phase (Chae et al., 2020). The ideal bone repair scaffold degradation and tissue repair curve is shown in Figure 10A. As time increases, the scaffold gradually degrades and the mechanical support curve decreases with time, while the curve of new tissue formation gradually increases with time, and the two complement each other, thus maintaining the mechanical support strength during the bone repair process. However, the majority of existing scaffolds have a changing mechanical support strength curve after implantation as shown in Figure 10B. On the one hand, the scaffold degrades quickly resulting in insufficient mechanical support for the healing tissue formed initially; on the other hand, the scaffold does not degrade or degrades too slowly, resulting in the growth of new bone tissue being impeded and a stress masking effect, leading to difficulties in providing sufficient mechanical support for the new bone once the mechanical properties of the scaffold are insufficient at a later stage, as shown in Figure 10C. As titanium-magnesium composites are partially degradable materials, only the surface Mg will be rapidly degraded after implantation into the body, the exposed porous structure of the titanium will provide mechanical support in the form of a skeleton. The degradation of Mg *in vivo* also serves to induce the formation of bone tissue, and the voids formed by the degraded Mg also provide space for bone tissue to grow. From this point of view, titanium-magnesium composites have an advantage over other human implants that cannot be matched.

**TABLE 1:** Corrosion properties of titanium-magnesium composites in simulated body fluids.

Sample	Solution	Corrosion current density (mA/cm^2^)	Corrosion potential (V)	References
Ti-10 Mg	SBF	0.00546 ± 0.00064	−0.499 ± 0.0232	Ouyang et al. (2020)
Ti-20 Mg	SBF	0.00349 ± 0.00052	−0.4289 ± 0.0148	Ouyang et al. (2020)
Ti-30 Mg	SBF	0.00359 ± 0.0036	−0.5046 ± 0.0126	Ouyang et al. (2020)
Ti-5Mg	SBF	0.0125	−0.970	Ouyang et al. (2020)
Ti-10 Mg	SBF	0.0258	−1.312	Lai et al. (2022)
Ti-20 Mg	SBF	0.0581	−1.427	Lai et al. (2022)
Ti-30 Mg	SBF	0.479	−1.469	Lai et al. (2022)
Ti6Al4V-Mg	Ringer’s	0.429 ± 0.12	−1.47 ± 0.30	Esen et al. (2020)
Ti6Al4V-AZ91	Ringer’s	0.367 ± 0.09	−1.44 ± 0.30	Esen et al. (2020)
Ti6Al4V-WE43	Ringer’s	0.381 ± 0.08	−1.45 ± 0.36	Esen et al. (2020)
Ti-50 Mg	Ringer’s	1.90	−1.472	Esen et al. (2013)
Ti-60 Mg	Ringer’s	1.57	−1.493	Esen et al. (2013)
Ti-70 Mg	Ringer’s	1.07	−1.490	Esen et al. (2013)
Ti-80 Mg	Ringer’s	1.12	−1.512	Esen et al. (2013)

**TABLE 2:** Conventional scale for assessing corrosion resistance (Tsakiris et al., 2021).

Resistance	i_corr_ (mA/cm^2^)	Stability group
Completely stable	<0.001	4
Very stable	0.001–0.01	3
Stable	0.01–0.1	2
Low stable	0.1–1	1
Unstable	>1	0

### Negative effects of corrosion and mitigation measures

The degradation of Mg and Mg alloys increases the OH^−^ concentration in the environment (Atrens et al., 2013), which can lead to an increase of pH around the local implant tissues and increase the risk of alkalosis in the human body. Esen et al. (2020) found that the pH of a variety of titanium-magnesium composites soaked in physiological solutions for up to 48 h increased dramatically to 10 or even higher. High pH can lead to severe internal injuries, but as mentioned earlier, the implant induces inflammation in the tissues surrounding the implant during the pre-implantation phase, creating an acidic environment where these acids may neutralize the OH^−^ produced by the titanium-magnesium composite, which is helpful for the body and the implant. In addition to the high pH associated with the degradation of Mg, Ti at the cathode precipitates large amounts of hydrogen gas (Cao et al., 2013). The accumulation of large amounts of this hydrogen in the patient’s body causes local discomfort rather than being harmful to the tissue. Over time, this hydrogen may be eliminated from the body or can be eliminated by a puncture procedure (Brooks et al., 2016). Although large amounts of hydrogen are not harmful to humans, they are extremely harmful to the properties and structure of the material. It has been found that excessive hydrogen pressure can exceed the self-strength of porous materials, causing the composite to lose its mechanical integrity and eventually face failure (Esen et al., 2013). The rapid corrosion rate of titanium-magnesium composites is a major factor limiting their use in human implants. Reducing the degradation of magnesium in composites is a pressing issue. Surface modification is considered to be the most widely used method to reduce the degradation rate of Mg-containing materials. Surface modification can be achieved by mechanical or chemical treatment. Chemical pre-washing can eliminate Mg from the composite surface in advance, thus exposing the porous structure, and this gradient composite has less effect on force properties (Jiang et al., 2015), but these surface pores can act as deep pockets for post-implantation bacterial infection (Weber and Cochran, 1998), and this method cannot be applied to mass production. Subsequently, Ibrahim et al. (2021) obtained flat, smooth and undamaged surfaces by mechanical treatment with grinding and polishing, and found that the degradation rate of mechanically treated titanium-magnesium composites was reduced by a factor of five compared to untreated ones. In addition, Xu et al. (2021) successfully generated a layer of oxide film on the surface of titanium-magnesium composites by micro-arc oxidation. Subsequently they found that the hydrogen precipitation rate of the micro-arc-oxidized titanium-magnesium composites was alleviated, and the degradation rate of the composites was significantly reduced. The microarc oxidation technique can not only reduce the corrosion rate of Mg phase in composites, but also stimulate the adhesion, value addition and differentiation of cells on Ti phase (Chen et al., 2019). Taken together, micro-arc oxidation technology has a positive effect on both Ti and Mg phases in titanium-magnesium composites, and it is one of the technologies with more outstanding advantages in the surface modification scheme of titanium-magnesium composites. The human body is a complex chemical environment, and factors such as inorganic ions, amino acids and oxidative stress reactions in the human body may affect the degradation behavior of magnesium. The effect of this environmental factor on the corrosion rate of titanium-magnesium composites has not been studied in depth, and these studies are necessary before titanium-magnesium composites are implanted into the human body as bio-implants.

## Conclusion and future perspectives

This work summarizes the recent progress of titanium-magnesium composites. Titanium-magnesium composites have shown significant promise for orthopedic applications. The titanium-magnesium composites have the following advantages:

1) The E of the composite may be modified across an extensive range by adjusting the titanium and magnesium composition ratios. In contrast, the inclusion of magnesium compensates for porous titanium’s probable lack of strength.
2) The addition of magnesium to the composites also addresses titanium’s lack of bioactivity.
3) Magnesium decomposition increases bone tissue formation and offers space for it to expand. The porous titanium in the composite can give long-term mechanical support for bone tissue growth.

The fabrication of partially degradable titanium-magnesium composites shows promise from a processing standpoint because all three preparation techniques successfully combined titanium and magnesium and displayed favorable properties that are either difficult to obtain or impractical through conventional melting or deformation processing. The mechanical properties of the currently prepared titanium-magnesium composites can meet the performance requirements of human implants, but researchers are still exploring the development of titanium-magnesium composites with lower E and higher UCS. Researchers are attempting to create titanium-magnesium composites with lower degradation rates from the perspective of composition selection, such as by lowering the surface volume share of magnesium in the composites and creating titanium-magnesium composites using either titanium alloys or magnesium alloys. Nevertheless, the partial degradability and post-degradation strength of Ti-Mg composites are advantageous for biological applications. However, research on titanium-magnesium composites is still in its early stages compared to that of titanium alloys, stainless steel, and cobalt-based alloys. Before titanium-magnesium composites may be used in biomedical domains, much more work needs to be done. In order to consistently and easily produce high-quality samples, novel preparation techniques should be investigated and implemented. Second, the current studies on the mechanical characteristics of titanium-magnesium composites primarily focus on the E test and few works looking into the compressive and tensile properties performance, while studies on fatigue testing have been noted to be insufficient. In addition, corrosion fatigue, a complicated relationship between mechanical and corrosion features in a body fluid environment, is still absent. Finally, the relationship of mechanical behavior-corrosive properties-porosity is not to be established.
